# Gut microbiota associated with cryptococcal meningitis and dysbiosis caused by anti-fungal treatment

**DOI:** 10.3389/fmicb.2022.1086239

**Published:** 2023-02-22

**Authors:** Hang Li, Lei Zhang, Keming Zhang, Yue Huang, Yi Liu, Xiaodi Lu, Wanqing Liao, Xiaogang Liu, Qilong Zhang, Weihua Pan

**Affiliations:** ^1^Shanghai Key Laboratory of Molecular Medical Mycology, Shanghai Changzheng Hospital, Second Military Medical University, Shanghai, China; ^2^Department of Dermatology, Shanghai Changzheng Hospital, Second Military Medical University, Shanghai, China; ^3^Department of Dermatology, The First Affiliated Hospital of Nanchang University, Nanchang, China; ^4^Department of Dermatology, The Third Affiliated Hospital of Xi’an Jiaotong University, Shaanxi Provincial People’s Hospital, Xi’an, China; ^5^Department of Dermatology, The First Naval Hospital of Southern Theater Command, Zhanjiang, China; ^6^Department of Neurology, Jiangxi Chest Hospital, Jiangxi, China

**Keywords:** cryptococcal meningitis, gut microbiota, metagenome, anti-fungal therapy, diagnosis

## Abstract

The gut microbiota is a dynamic and highly diverse microbial ecosystem that affects many aspects of the host’s physiology. An improved understanding of the gut microbiota could lead to better strategies for the diagnosis and therapy of cryptococcal meningitis (CM), but the impact of *Cryptococcus* infection and anti-fungal treatment on the gut microbiota has rarely been studied. We characterized the diversity and composition of the gut microbiota in CM patients at diagnosis and healthy controls (HCs) using metagenomic sequencing and determined the effects of anti-fungal drugs. We found that CM patients had distinct bacterial and fungal compositions compared with HCs, with eight differentially abundant fungal and 72 differentially abundant bacterial species identified between the two groups. CM patients showed an increased abundance of *Enterococcus avium*, *Leuconostoc mesenteroides*, and *Weissella cibaria*, and a decreased abundance of *Prevotella* spp. compared with HCs. However, anti-fungal treatment only led to minor changes in the intestinal microbiota. Moreover, both positive and negative correlations existed in fungal, bacterial, and clinical indicators. Our study suggests that the *Cryptococcus neoformans* infection caused a distinct dysbiosis of the gut microbiota and contributes valuable information implying potential links between the CM and gut microbiota.

## Introduction

Cryptococcal meningitis (CM) is a global invasive fungal disease associated with high morbidity and mortality (Iyer et al., 2021; Rajasingham et al., 2022). *Cryptococcus neoformans* (*C. neoformans*) and *C. gattii* are the main species responsible for life-threatening CM (Hagen et al., 2015; Kwon-Chung et al., 2017); immunocompromised individuals are the most vulnerable, but there are also reports of cryptococcal infections in immunocompetent hosts (Perfect et al., 2010; Kwon-Chung et al., 2014). It is estimated that 223,100 cases of CM occur globally each year, leading to 181,100 deaths (Rajasingham et al., 2017). Most CM-related deaths occur in resource-limited settings in which access to drugs is limited and effective treatments are expensive, signifying the need to develop affordable therapeutics against these deadly fungal pathogens (Loyse et al., 2019; Vilas-Bôas et al., 2020; Driemeyer et al., 2022; Rajasingham et al., 2022).

Recent increases in our understanding and analysis of the gut microbiota have shed light on the impact of alterations to human health. The gut microbiota is a dynamic and highly diverse microbial ecosystem that affects many aspects of the host’s physiology (Kamada et al., 2013; Erny et al., 2021), and is involved in the host immune response, protection against pathogen overgrowth, biosynthesis, and metabolism. For example, the microbiota was found to be important for the host response against *C. gattii*, with germ-free mice more susceptible to infection, and showing lower survival, higher fungal burden in the lungs and brain, reduced levels of interferon-γ, interleukin (IL)-1β, and IL-17, and lower nuclear factor κB p65 phosphorylation than wild-type mice (Costa et al., 2016). Additionally, bloodborne *Candida albicans* infection was reported to decrease the diversity of the gut microbiota (Hu et al., 2021), while the gut bacterial microbiota affected generation of the pulmonary IL-17 response to *Aspergillus fumigatus* infection in mice (McAleer et al., 2016).

These findings highlight the commensal microbiota modules immune responses in infectious diseases and show that altering the composition could be a therapeutic approach for disease. However, it is unclear whether and how *Cryptoccocus* infection induces changes in the gut microbiota in human. In particular, the effect of anti-fungal treatments on the gut microbiota is unknown. Previous studies primarily focused on anti-bacterial and anti-viral treatment (Villanueva-Millán et al., 2017; Wipperman et al., 2017; Ponziani et al., 2018), but little is known about the effects of anti-fungal treatment on the intestinal microbiome. Additionally, most earlier studies on the gut microbiota concentrated on the bacterial community, while research into gut fungal communities only started more recently. Thus, more comprehensive research into the role of intestinal bacteria and fungi in the pathobiology of fungal diseases is needed.

To this end, we performed a cross-sectional study of CM patients and healthy controls (HCs) to characterize the bacterial and fungal gut microbiota changes in response to *Cryptococcus* infection and anti-fungal therapy in individuals from China.

## Materials and methods

### Study population

Patients with CM were recruited from Jiangxi Chest Hospital. A diagnosis of CM was based on positive results from cerebrospinal fluid (CSF) culture, a CSF cryptococcal antigen test (IMMY, Norman, OK, USA), or positive CSF India ink staining (Perfect et al., 2010). Inclusion criteria were a confirmed CM patient; age ≥ 18 years; and body mass index (BMI) between 18 and 25 kg/m^2^. Exclusion criteria were anti-fungal therapy administered prior to admission; recurrent CM; a known history of autoimmune or rheumatic diseases, metabolic diseases, chronic gastrointestinal diseases, or malignant tumors; pregnant or breastfeeding; presence of acute infection; a history of gastrointestinal tract surgery; and intake of antibiotics or colon-cleansing preparation within the last 3 months preceding stool collection. HCs were recruited *via* the healthy donor biobank of the Neurology Department in Jiangxi Chest Hospital.

Participants were divided into four groups: HCs (*n* = 16), CM patients not receiving therapy (CM group; *n* = 15), CM patients receiving 2 weeks of anti-fungal therapy (CM-2W group; *n* = 7), and CM patients receiving 4 weeks of anti-fungal therapy (CM-4W group; *n* = 6). All subjects were HIV-negative. This research was approved by the Institute Ethics Committee of Jiangxi Chest Hospital. All participants provided their written informed consent before stool donation.

### Collection of clinical data

Clinical data were collected by reviewing medical charts. The following data were recorded: age, sex, BMI, clinical manifestations like headache, fever, nausea, and vomiting, conscious disturbance and other neurological symptoms such as those associated with vision and hearing, CSF examination, treatment, and outcome.

### Stool collection and DNA extraction

Whole stools were collected in sterile boxes, immediately homogenized, and aliquots of 200 mg were frozen at –80°C for further analysis. Genomic DNA was extracted from aliquots using the QIAamp PowerFecal Pro DNA Kit (Qiagen, Germantown, MD, USA) according to the manufacturer’s instructions. The DNA pellet was resuspended in 80 mL of trypsin–ethylenediaminetetraacetic acid buffer, and the concentration was measured using the Qubit™ dsDNA Assay Kit with the Qubit™ 3.0 Fluorometer (Thermo Fisher Scientific, Carlsbad, CA, USA).

### Library construction and shotgun metagenomic sequencing

A total of 200 ng DNA was used as input material for sample preparations. Sequencing libraries were generated using the KAPA HyperPlus Library Preparation Kit (Roche, Basel, Switzerland) following the manufacturer’s recommendations, and index codes were added to attribute sequences to each sample. Briefly, the DNA sample was fragmented to a size of 350 bp, then fragments were end-polished, A-tailed, and ligated with the full-length adaptor for Illumina sequencing with further PCR amplification. Finally, PCR products were purified using the AMPure XP system, and libraries were analyzed for size distribution by the Agilent 2100 Bioanalyzer system (Agilent, Santa Clara, CA, USA) and quantified using real-time PCR. Whole-genome shotgun sequencing was performed on the NovaSeq 6000 system (Illumina, San Diego, California, USA) to obtain paired-end reads with 150 bp in the forward and reverse directions. Each sample obtained an average of 8G raw data.

### Statistical analyses

Participant characteristics are expressed as medians (ranges) and were compared using Mann–Whitney or χ^2^-tests as appropriate. Prism v.9.0 software (GraphPad, San Diego, CA, USA) was used for analyses and graph preparation. Alpha diversity, reflecting the species richness and diversity, was measured using the Pielou index, number of genes, and Shannon index. Beta diversity, comparing the similarity of species diversity among groups, was calculated using principal coordinate analysis (PCoA). A heatmap was drawn of the relative abundance of bacteria and fungi based on data from the Wilcoxon rank sum test adjusted by the Benjamini–Hochberg procedure (FDR < 0.1, fold-change > 2). Correlation coefficients of observations among fungi, bacteria, and clinical indicators were calculated using Bland and Altman. *P*-values were adjusted by the Benjamini–Hochberg procedure. Differences with *P* < 0.05 were considered significant.

## Results

### Participant characteristics

A total of 16 CM patients and 15 HCs were recruited to the study. CM patients received the same anti-fungal therapy regime. Treatment involves induction with amphotericin B in combination with flucytosine for 2 weeks, followed by consolidation and maintenance with fluconazole. There were no significant differences between CM patients and HCs with respect to age, sex, or BMI. One CM patient died before their 4-week treatment period was complete. Detailed demographic and clinical characteristics are shown in Table 1.

**TABLE 1:** Demographic features of CM patients and healthy controls.

Parameter	CM patients (*n* = 15)	HC (*n* = 16)	*P*-value
Age (years)	46.67 ± 15.94	43.25 ± 14.32	0.535
Sex: Male	9 (60%)	8 (50%)	0.576
Body mass index (kg/m^2^)	21.10 ± 0.84	21.63 ± 1.16	0.158
Clinical parameters			
Headache (*n* %)	13 (86.67%)	–	–
Fever (*n* %)	9 (60.00%)	–	–
Nausea/Vomiting (*n* %)	10 (66.67%)	–	–
Vision disorder (*n* %)	4 (26.67%)	–	–
Auditory symptoms (*n* %)	1 (6.67%)	–	–
Conscious disturbance (*n* %)	5 (33.33%)	–	–
CSF examination			
Opening pressure (mmH_2_O)	330.00 (360.00–180.00)	–	–
CSF protein (mg/l)	823.80 (1001.50–535.40)	–	–
CSF glucose (mmol/l)	1.80 ± 1.06	–	–
Treatment			
AMB + 5FC (*n* %)	15 (100%)	–	–
Fluconazole (*n* %)	15 (100%)	–	–
In-hospital mortality (*n* %)	1 (6.67%)	–	–
	

CM, cryptococcal meningitis; HC, healthy control; AMB, amphotericin B; FC, flucytosine.

### Bacterial dysbiosis in CM

**FIGURE 1:**
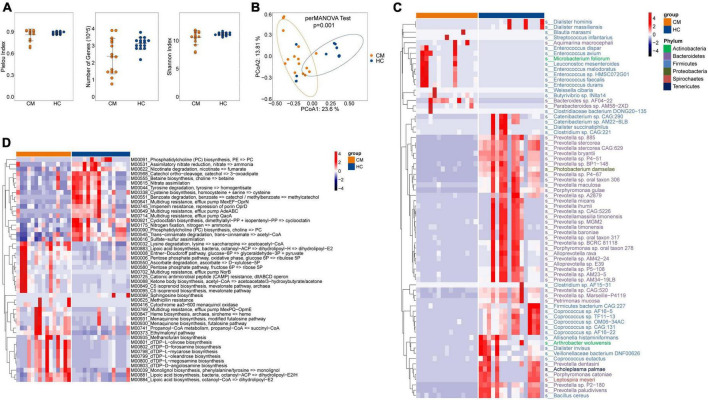
Comparisons of the structure and composition of bacteria between CM patients and HCs. **(A)** Alpha diversity analysis plot at the gene level, including the number of genes, and the Shannon index. Dot plots show the comparison between CM patients and healthy controls. **(B)** Principal component analysis plot based on the Bray–Curtis distance. **(C)** Heatmap of the relative abundance of bacteria. **(D)** Heatmap of the relative abundance of KEGG modules of bacteria. CM, cryptococcal meningitis; HC, healthy control.

No significant difference was observed in alpha diversity (assessed using three different indexes) between CM patients and the HC group (Figure 1A). However, the bacterial compositions of CM patients and HCs were segregated on PCoA, with significant differences in beta diversity identified between the bacteria of the CM group and those of the HC group (Figure 1B, *P* < 0.001).

Next, we focused on comparisons at the species level, and identified 72 differentially abundant bacterial species between the two groups (*P* < 0.05) (Figure 1C). CM patients were significantly enriched in 16 species, including *Enterococcus avium*, *Leuconostoc mesenteroides*, and *Weissella cibaria* belonging to the Firmicutes phylum; *Aquimarina macrocephali*, *Bacteroides* sp. *AF04-22*, and *Parabacteroides* sp. *AM58-2XD* belonging to the Bacteroidete*s* phylum; and *Microbacterium foliorum* of the Actinobacteria phylum. The HC group was significantly enriched in *Prevotella s*p. 885, *Prevotella bryantii*, and *Prevotella micans* belonging to the Bacteroidete*s* phylum, and *Photobacterium damselae* of the Proteobacteria phylum.

We further constructed gene modules exhibiting significant enrichment in terms of Kyoto Encyclopedia of Genes and Genomes (KEGG) pathways, gene ontology (GO) categories, or hallmark gene sets. We identified 48 differentially abundant modules between CM patients and HCs. Twenty-eight pathways were significantly upregulated in CM patients compared with HCs (Figure 1D), including lysine degradation (M00032), lipoic acid biosynthesis (M00883), and pentose phosphate pathways (M00006). Phosphatidylcholine (PC) biosynthesis (M00091), tyrosine degradation (M00044), multidrug resistance (M00641), and nitrogen fixation (M00175) pathways were significantly upregulated in the HC group compared with CM patients.

### Altered fungal microbiota diversity in CM patients

**FIGURE 2:**
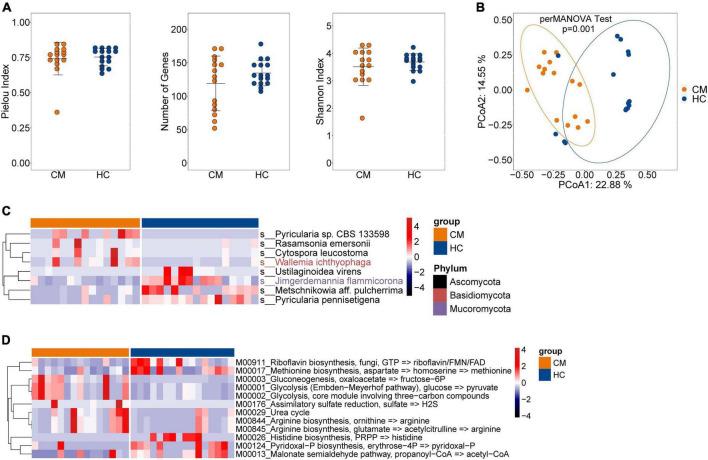
Comparisons of the structure and composition of fungi between CM patients and HCs. **(A)** Alpha diversity analysis plot at the gene level, including the number of genes, and the Shannon index. Dot plots show the comparison between CM patients and healthy controls. **(B)** Principal component analysis plot based on the Bray–Curtis distance. **(C)** Heatmap of the relative abundance of fungal. **(D)** Heatmap of the relative abundance of KEGG modules of fungal. CM, cryptococcal meningitis; HC, healthy control.

We next assessed the composition of the fungal microbiota in CM patients and HCs. Like bacteria, no significant difference was observed in alpha diversity between CM patients and HCs (Figure 2A), but a significant difference in beta diversity was detected (Figure 2B).

The fungal composition was then compared at the species level, and eight differentially abundant fungi were identified (Figure 2C). The CM group was enriched with *Pyricularia* sp. CBS 133598, *Rasamsonia emersonii*, and *Cytospora leucostoma* belonging to the Ascomycota phylum, and *Wallemia ichthyophaga* of the Basidiomycota phylum. *Ustilaginoidea virens*, *Metschnikowia aff. pulcherrima*, and *Pyricularia pennisetigena* belonging to the Ascomycota phylum, and *Jimgerdemannia flammicorona* of the Mucoromycota phylum were more abundant in HCs than CM patients.

We also identified 12 differentially abundant modules between CM patients and HCs (Figure 2D). Seven modules, including gluconeogenesis (M00003), glycolysis (M00001 and M00002), assimilatory sulfate reduction (M00176), urea cycle (M00029), and arginine biosynthesis (M00844 and M00845) were significantly enriched in CM patients compared with HCs. Riboflavin biosynthesis (M00911), methionine biosynthesis (M00017), histidine biosynthesis (M00026), pyridoxal-P biosynthesis (M00124), and the malonate semialdehyde pathway (M00013) exhibited higher abundance in the HC group compared with CM patients.

### Anti-fungal treatment induces minor alterations in the bacterial and fungal microbiota

**FIGURE 3:**
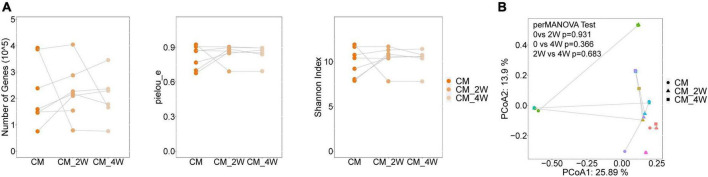
Comparison of the structure and composition of bacterial and fungal among HC, CM, and CM treatment groups. **(A)** Boxplot shows the mean distance of CM, CM_2W, and CM_4w to the HC group on the left. Principal component analysis plot of bacteria based on the Bray–Curtis distance is shown on the right. **(B)** Boxplot shows the mean distance of CM, CM_2W, and CM_4w to the HC group on the left. Principal component analysis plot of fungal based on the Bray–Curtis distance is shown on the right. HC, healthy control; CM, cryptococcal meningitis; CM-2W, cryptococcal meningitis patients receiving 2 weeks of anti-fungal therapy; CM-4W, cryptococcal meningitis patients receiving 4 weeks of anti-fungal therapy.

**FIGURE 4:**
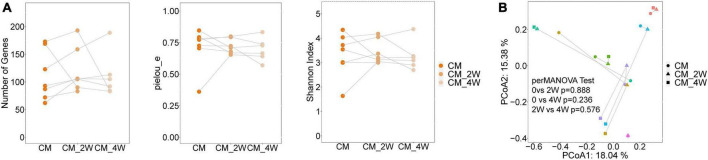
Changes of the structure and composition of bacteria during treatment in CM patients. **(A)** Alpha diversity analysis plot at the gene level, including the number of genes, and the Shannon index. Dot plots show changes before and after treatment. Dots connected by the gray line represent individuals. **(B)** Principal component analysis plot based on the Bray–Curtis distance. Individuals are plotted by the same color and connected by the gray line. CM, cryptococcal meningitis; CM-2W, cryptococcal meningitis patients receiving 2 weeks of anti-fungal therapy; CM-4W, cryptococcal meningitis patients receiving 4 weeks of anti-fungal therapy.

**FIGURE 5:**
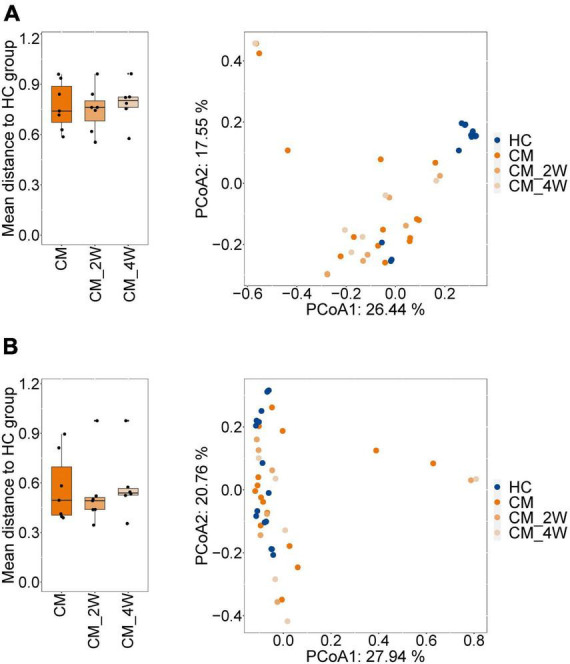
Changes of the structure and composition of fungal during treatment in CM patients. **(A)** Alpha diversity analysis plot at the gene level, including the number of genes, and the Shannon index. Dot plots show changes before and after treatment. Dots connected by the gray line represent individuals. **(B)** Principal component analysis plot based on the Bray–Curtis distance. Individuals are plotted by the same color and connected by the gray line. CM, cryptococcal meningitis; CM-2W, cryptococcal meningitis patients receiving 2 weeks of anti-fungal therapy; CM-4W, cryptococcal meningitis patients receiving 4 weeks of anti-fungal therapy.

Next, we examined the effects of anti-fungal treatment on the gut microbiota in CM patients. We compared the structure and composition of bacteria and fungi among HC, CM, and CM treatment groups. The boxplots show no significant difference in fungi and bacteria between groups (Figure 3). Then we analyzed individual participants separately using alpha diversity analysis at the gene level, including the number of genes, and the Shannon index. Dot plots show the changes before and after treatment. With anti-fungal drugs, the richness of bacteria and fungi in most individuals was shown to increase (Figures 4A, 5A). We then used PCoA to compare groups of samples, but clustering driven by CM, CM-2, and CM-4 groups was not significant (Figures 4B, 5B).

### Correlation network of fungi, bacteria, and clinical indicators

**FIGURE 6:**
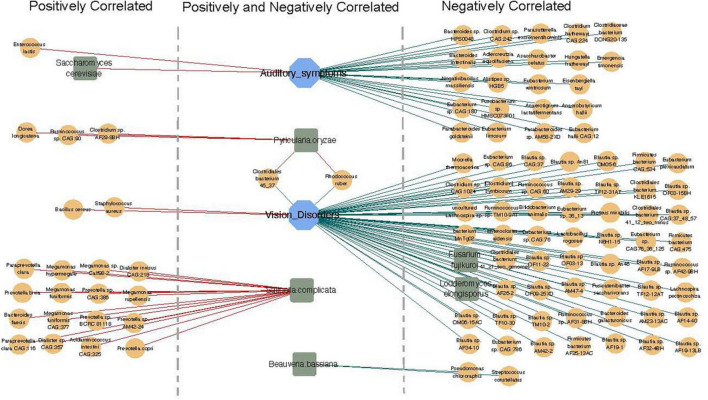
Correlation network of fungi, bacteria, and clinical indicators. Orange circle represents bacteria (relative abundance > 0.01%), blue square represents clinical indicators, and green hexagon represents fungi. Red lines show positive correlations and green lines show negative correlations.

Finally, we examined possible correlations between fungi, bacteria, and clinical indicators. We completed further correlation analysis using the Bland–Altman plot to identify co-occurring clusters of bacterial and fungal species. Both positive and negative correlations were shown to exist between fungi, bacteria, and clinical parameters (Figure 6). We also identified significant correlations between the gut microbiota and CM-related symptoms, including auditory symptoms and visual disorders. Auditory symptoms had a positive correlation with the abundance of *Enterococcus lactis* and *Saccharomyces cerevisiae*, and a strong negative correlation with 22 species of bacteria, including *Bacteroides* sp. *HPS0048*, *Clostridium* sp. *CAG:242*, and *Adlercreutzia equolifaciens.* Visual disorders showed positive correlations with two species of bacteria (*Bacillus cereus* and *Staphylococcus aureus)*, and negative correlations with 56 species of bacteria and two species of fungi.

Positive and negative correlations were also found to exist between bacteria and fungi. A strong positive correlation was detected between the abundance of *Pyricularia oryzae* with three species of bacteria (*Dorea longicatena*, *Ruminococcus* sp. *CAG:90*, and *Clostridium* sp. *AF29-8BH).* Moreover, *Saitoella complicata* was positively correlated with 16 species of bacteria, including *Paraprevotella clara, Megamonas hypermegale*, and *Prevotella bivia*, while *Beauveria bassiana* was negatively correlated with *Pseudomonas chlororaphis* and *Streptococcus constellatus* (Figure 6).

## Discussion

Since the importance of microbiota was established, numerous studies have revealed associations between the gut microbiota and human disease (Qian et al., 2021; Trebicka et al., 2021; Wu et al., 2021). Dysbiosis of the gut microbiota during *Cryptococcus* infection or anti-fungal treatment may play an important role in the pathophysiology of CM. To investigate this, we examined the gut microbiota of CM patients with and without anti-fungal treatment and healthy subjects using shotgun metagenomic sequencing. Our results revealed that CM patients have distinct bacterial and fungal compositions compared with controls, but that anti-fungal treatment only led to minor changes in the gut microbiota. Moreover, both positive and negative correlations existed between fungi, bacteria, and clinical indicators in CM. Our study contributes valuable information to the field about the gut microbiota in CM patients, implying the existence of links between gut microbiota changes and CM.

We showed that the gut microbiota composition of CM patients differed significantly from that of healthy individuals with respect to beta diversity, but not alpha diversity. This contrasts with previous findings which revealed the alpha diversities of gut microbiota to be reduced in patients with infectious diseases compared with controls (Wang et al., 2017; Hu et al., 2019; Tuddenham et al., 2020).

*Enterococcus* is a genus of Gram-positive lactic acid bacteria of the Firmicutes phylum. Some enterococcal strains cause antibiotic-induced biological disorders, play antitumor or anticancer roles, and modulate the immune system. For example, cultured *E. faecium* from the human intestinal epithelium demonstrated bactericidal effects against enteroaggregative *Escherichia coli*, as well as membrane damage, and cell lysis (Tarasova et al., 2010; Fusco et al., 2017), while in another study it increased the expression of proinflammatory cytokines without appearing as a pathogen (Seishima et al., 2019). The present study observed significant increases in the abundance of six *Enterococcus* species in CM patients compared with controls, including *E. dispar*, *E. avium*, and *E. faecalis*. Considering the beneficial actions of *Enterococcus* bacteria on the host immune system, it seems that *Cryptococcus* infection-induced increases in the abundance of bacteria reflect a compensatory response in the host.

We also found that CM patients were significantly enriched in *Blautia marasmi.* The *Blautia* genus of the Lachnospiraceae family in the Firmicutes phylum was initially described in 2008 (Liu et al., 2008). *Blautia* species function in the degradation of indigestible carbohydrates (Sheridan et al., 2016), and some such as *B. coccoides* produce short-chain fatty acids as metabolic mediators between the microbiota and host (Liu et al., 2021). Considering its relatively high abundance in CM patients, *B. marasmi* may play an important role in *Cryptococcus* infection.

We observed a significant decrease in the abundance of *Prevotella* spp. in the CM group compared with controls. *Prevotella* is a diverse genus of Gram-negative anaerobes, which is highly abundant in different parts of the body and plays a key role in the balance between health and disease (Shah and Collins, 1990; Larsen, 2017; Posteraro et al., 2019). Conflicting findings have been reported about whether *Prevotella* spp. are beneficial or detrimental to gut health, especially with regard to glucose homeostasis (Ley, 2016; Cani, 2018; Claus, 2019). The emergence of *Prevotella*-rich microbiota is associated with inflammatory disorders such as rheumatoid arthritis, periodontitis, metabolic disease, and inflammation in HIV patients (Ibrahim et al., 2017; Armstrong et al., 2018; Wells et al., 2020). Several studies have further demonstrated the potential pro-inflammatory role of *Prevotella* spp., including enhanced T cell activation and T cell recruitment (Neff et al., 2018; Rolhion et al., 2019; Wells et al., 2020). Considering the important role of T cell immunity in preventing *C. neoformans* infection, the observed decrease in *Prevotella* abundance in CM patients of the present study may represent one of the mechanisms of *C. neoformans* against host immunity.

KEGG is a widely used annotated database (Kanehisa et al., 2004). Genes can be projected into the KEGG PATHWAY database uncover interactions with other genes that may influence the health of the host (Altermann and Klaenhammer, 2005). our data showed that gluconeogenesis, glycolysis and lipoic acid biosynthesis much more abundant in CM groups. Some studies have found that gluconeogenesis, glycogenolysis, and glycolytic pathways produce glucose-6-phosphate and further releases abundant nicotinamide adenine dinucleotide phosphate through the pentose phosphate pathway (Agius et al., 2002; Gomis et al., 2003). High levels of glutathione promote inflammatory macrophages to mediate inflammatory responses. Additionally, glycogen metabolism not only upregulates STAT1 expression by activating RARβ but also promotes STAT1 phosphorylation by downregulating phosphatase TC45 in macrophage, thereby regulating inflammatory responses (Ma et al., 2020). Therefore, alterations in the function of the gut microbiota may play an important role in the pathogenesis of CM. In our study, lipoic acid biosynthesis much more abundant in CM groups, lipoic acid is an antioxidant that has been suggested to have beneficial immunomodulatory effects on the innate and adaptive immune systems in autoimmune diseases (Liu et al., 2019). Further investigations are needed to better understand the relationship between different features of the gut microbiota in CM.

Our results showed that anti-fungal treatment induced only a minor alteration in the gut microbiota, with the clustering driven by CM, CM-2, CM-4 groups not reaching significance based on PCoA analysis. The effect of anti-infection treatments on the gut microbiota is unclear. Antibiotics, especially broad-spectrum antibiotics, can destroy the gut microbiota during anti-tuberculosis (TB) treatment in mice (Namasivayam et al., 2017). However, a human study by Wipperman et al. (2017) reported no significant difference in the overall microbiota diversity of active TB patients after using first-line drugs compared with uninfected or latent TB patients, as estimated by the Shannon diversity index. Moreover, anti-infection treatments, especially anti-viral treatment for non-intestinal infections, may help restore the intestinal flora. For example, it was reported that a combination of nucleoside reverse transcriptase inhibitors reduced the fecal bacterial diversity caused by HIV infection (Villanueva-Millán et al., 2017). However, a similar restoration of the intestinal flora was not seen in our study.

Fungi and bacteria share microhabitats and participate in complex communications within the microbial community. The correlation network of fungi and bacteria observed in our study shows these co-occurrence patterns in CM patients after treatment. The bacterium *S. constellatus* showed a negative correlation with the fungus *B. bassiana*, *Streptococcus* is the most common genus of bacteria present in patients with bacterial brain abscesses and is often isolated from mixed infections. *S. stellate* typically resides in the oral cavity, appendix, and female reproductive tract, and tends to form abscesses (Lanks et al., 2019). Our study found negative correlation between *S. constellatus* and *B. bassiana*, and this negative relationship need confirmed in larger cohorts.

There are several limitations of this study, including its relatively small sample size, which may have resulted in a lack of power for some comparisons, especially in post-treatment patients. Future studies should therefore expand the sample size. Additionally, the lack of follow-up prevented a longitudinal analysis. Moreover, the mechanisms underlying increases in the abundance of bacteria in the gut of mice with *Cryptococcus* infections should be investigated.

## Conclusion

In conclusion, this study shows that *Cryptococcus* infection induces significant changes in the gut microbiota, with CM patients showing distinct bacterial and fungal compositions compared with controls. Both positive and negative correlations were found to exist between fungal and bacterial species after treatment. These data serve as a basis for further investigations into the role of the gut microbiome in CM patients.

## Data availability statement

The Metataxonomic raw data have been deposited in the NCBI online repository under the accession number PRJNA935276.

## Ethics statement

The studies involving human participants were reviewed and approved by the Institute Ethics Committee of Jiangxi Chest Hospital. The patients/participants provided their written informed consent to participate in this study.

## Author contributions

HL and LZ performed the experiments and wrote the manuscript. KZ, YH, XDL, and YL contributed to the methodology and helped with data analysis. WL, XGL, WP, and QZ contributed to the study design and modified the manuscript for final submission. All authors contributed to the article and approved the submitted version.
